# RNA virome comparison between sylvatic and urban-interface mosquitoes from Southeastern Brazil

**DOI:** 10.3389/fcimb.2026.1894867

**Published:** 2026-09-04

**Authors:** Geovani de Oliveira Ribeiro, Lilian de Oliveira Guimarães, Endrya do Socorro Foro Ramos, Roseane da Silva Couto, Juliana Telles-de-Deus, Vanessa Christe Helfstein, Vanessa dos Santos Morais, Simone Luchetta Reginato, Luis Filipe Mucci, Eduardo Sterlino Bergo, Ramendra Pati Pandey, Vera Lucia Fonseca de Camargo-Neves, Antonio Charlys da Costa, Élcio Leal, Karin Kirchgatter

**Affiliations:** 1 Leônidas and Maria Deane Institute (ILMD), Fiocruz Amazônia, Oswaldo Cruz Foundation, Manaus, Brazil; 2 Pasteur Institute, São Paulo, Brazil; 3 Viral Diversity Laboratory, Institute of Biological Sciences, Federal University of Pará, Belém, Brazil; 4 School of Biosciences and Bioengineering, D. Y. Patil International University, Pune, India; 5 Institute of Tropical Medicine, University of São Paulo, São Paulo, Brazil

**Keywords:** Culicidae, host–virus interactions, insect-specific viruses, metagenomics, mosquito virome, neotropical region, sylvatic–urban gradient, viral discovery

## Abstract

**Introduction:**

Mosquitoes (Diptera: Culicidae) are primary vectors of public health pathogens, yet their core viromes remain poorly characterized, particularly in Neotropical sylvatic lineages. This study investigated the RNA virome of multiple mosquito species across urban-to-forest gradients in São Paulo State, Brazil, including neglected sylvatic taxa such as *Sabethes, Psorophora, Shannoniana*, and *Wyeomyia*.

**Methods:**

The RNA virome of multiple mosquito species was investigated across urban-to-forest gradients in São Paulo State, Brazil. Ecological analyses were performed to assess the effects of host taxonomy and environment on virome composition. Network analysis was conducted to investigate virus–host associations and viral sharing across ecological interfaces.

**Results:**

Our analysis identified 919 viral contigs across 217 viral species and 37 distinct families, revealing a substantial fraction of “viral dark matter” with low amino acid identity (median < 40%) in predominantly sylvatic mosquito species. Although viral families containing known arboviruses, such as *Flaviviridae, Phenuiviridae,* and *Peribunyaviridae*, were detected, no high-consequence human pathogens were identified within the sensitivity limits of our sampling and sequencing depth. Ecological analyses demonstrated that virome composition was strongly structured by host taxonomy and environment (R^2^=0.570, *p*=0.001), with host species explaining 32.9% of the unique variance (PERMANOVA, R^2^=0.329, *p*=0.001), whereas ecotope played a secondary role (R^2^=0.029, p=0.001). Network analysis revealed a highly modular virus–host structure dominated by host-restricted specialists, with a limited number of bridge species facilitating viral sharing across ecological interfaces.

**Discussion:**

These findings indicate that intrinsic mosquito biology is the main driver of viral community structure, while environmental gradients play a secondary role, and highlight the importance of host-associated processes in shaping viral diversity at the Neotropical forest–urban interface.

## 1 Introduction

Mosquitoes *Culicidae* family constitute one of the most ecologically diverse groups of hematophagous insects and play central roles in the maintenance, amplification, and transmission of high-impact public health arboviruses, such as dengue, Zika, chikungunya, and yellow fever viruses. However, these medically relevant agents represent only a fraction of the total viral diversity associated with these insects. The complete set of viruses present in mosquitoes is known as the virome, which encompasses insect-specific viruses (ISVs), plant-associated and fungal-associated viruses, as well as a vast diversity of as-yet unclassified RNA viruses. Many of these components are not directly related to human diseases but integrate the viral microbiota and play structural roles in microbial ecology and evolution (Nebbak et al., 2021; Liu et al., 2023; Kacnik et al., 2025).

Brazil, characterized by intense urbanization coupled with high biodiversity, provides a valuable scenario to investigate how ecological gradients influence the structuring of mosquito-associated viral communities. The Southeastern region, specifically the state of São Paulo, brings together densely urbanized areas, peri-urban environments, and preserved forest ecosystems, allowing for comparative analyses across different levels of anthropization (Guimarães et al., 2024; Gómez-Palacio et al., 2025).

The composition of the viral microbiota varies markedly according to the ecological niche occupied by different mosquito species. Anthropized and peri-urban environments: Genera such as *Aedes* (e*.g., Ae. aegypti and Ae. aenigmaticus)* and *Culex (Cx. quinquefasciatus, Cx. chidesteri, Cx. renatoi*) stand out, as they are frequently adapted to artificial breeding sites and densely populated areas. These genera are recognized arbovirus vectors and tend to harbor viral microbiotas influenced by the urban context and proximity to human hosts (Christie et al., 2023; Lim et al., 2025). The genus *Anopheles* (*e.g., An. strodei*), although more associated with rural and peri-urban environments, also participates in microbial dynamics within modified landscapes. In contrast, predominantly forest-dwelling genera such as *Haemagogus* (*Hg. leucocelaenus), Sabethes (Sa. aurescens, Sa. purpureus, Sa. identicus, Sa. albiprivus), Shannoniana (Sh. fluviatilis), Psorophora (Ps. albigenu, Ps. varipes), Mansonia (Ma. humeralis)*, and *Wyeomyia (Wy. confusa*) are associated with natural breeding sites and sylvatic ecological cycles. These genera sustain enzootic cycles and act as reservoirs for a broad viral diversity, which is fundamental for understanding spillover events at the wildlife-human interface (Weaver et al., 2018; Childs et al., 2019; Hernández-Villegas et al., 2025).

Viral diversity is strongly modulated by the phylogenetic proximity between hosts and the environmental context. Phylogenetically related species tend to share subsets of viruses, while more distantly related species exhibit highly specialized viromes, reflecting ecological filtering processes and host-associated restrictions (Pan et al., 2024). Furthermore, factors such as feeding habits, trophic breadth, and breeding site characteristics directly influence exposure to environmental and plant viruses, shaping the architecture of the viral microbiota (Liu et al., 2023).

ISVs, often predominant in these systems, can modulate vector competence and interfere with the replication of pathogenic arboviruses, highlighting complex interactions within the viral microbiota (Konstantinidis et al., 2022b). Bipartite virus–host network approaches reveal high modularity and specificity, suggesting that viral persistence and sharing are regulated by host intrinsic biological factors and local ecological structure (Konstantinidis et al., 2022b).

Advances in metagenomics and next-generation sequencing (NGS) have substantially expanded our knowledge of the viral diversity within the mosquito microbiota across different regions of the world (Liu et al., 2023; Hernández-Villegas et al., 2025; Kacnik et al., 2025). These studies demonstrate that the virome is highly dynamic and strongly influenced by environmental pressures, mosquitoes species, ecological interactions, and local trophic webs (Nebbak et al., 2021; Hermanns et al., 2023). Thus, mosquitoes do not act merely as passive vectors, but as ecological filters that shape microbial composition through host physiological restrictions, viral compatibility, and evolutionary processes associated with host–virus coadaptation (Pan et al., 2024).

Despite recent advances, significant gaps persist in Neotropical regions, especially regarding predominantly sylvatic genera such as *Sabethes, Psorophora, Shannoniana*, and *Wyeomyia*. Thus, the virome does not constitute a random assembly of viruses but results from the interaction between the host’s evolutionary history and the ecological context in which it is embedded, being structured by environmental filters and host-associated constraints.

In this study, we characterize the RNA virome associated with the microbiota of multiple mosquito species collected across urban, peri-urban, and forest gradients in southeastern Brazil. We sought to: (i) evaluate the diversity of the detected viruses; (ii) investigate the effect of the host taxonomy and ecological context on virome composition; and (iii) explore patterns of viral sharing and ecological modularity.

## 2 Methods

### 2.1 Mosquito collection and identification

**Figure 1:**
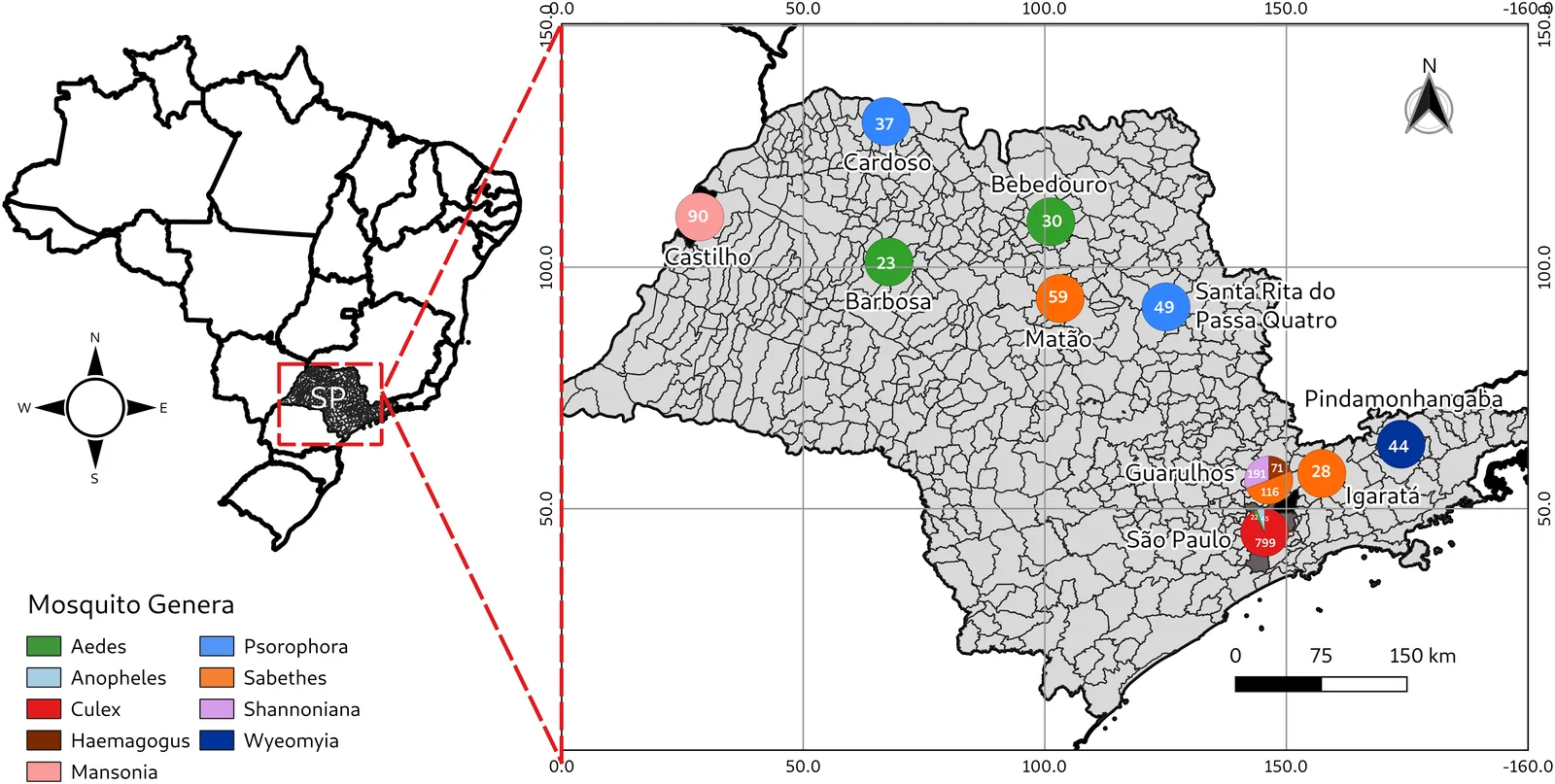
Spatial distribution of mosquito collection and analysis points by municipality. The points highlighted on the map correspond to the collection sites in each municipality during the sampling period. Each point is color-coded to represent the mosquito genera identified at each sampling site: green (*Aedes*), light green (*Anopheles*), red (*Culex*), brown (*Haemagogus*), pink (*Mansonia*), light blue (*Psorophora*), orange (*Sabethes*), lilac (*Shannoniana*), and dark blue (*Wyeomyia*). The numerical values associated with each point indicate the number of samples analyzed.

A total of 1604 mosquitoes belonging to the family *Culicidae* were collected, representing multiple genera. Taxonomic identification was performed based on morphological characters and taxonomic keys for Neotropical Culicidae, yielding a representative set of 17 species: *Aedes aegypti*, *Aedes aenigmaticus*, *Aedes scapularis, Anopheles strodei*, *Culex chidesteri*, *Culex quinquefasciatus*, *Culex renatoi*, *Haemagogus leucocelaenus*, *Mansonia humeralis*, *Psorophora albigenu*, *Psorophora varipes*, *Sabethes albiprivus*, *Sabethes aurescens*, *Sabethes identicus*, *Sabethes purpureus*, *Shannoniana fluviatilis*, and *Wyeomyia confusa*. Mosquitoes were collected in two distinct ecological contexts in the state of São Paulo, Brazil: (i) forested environments, including preserved forest and forest-edge areas, and (ii) an urban–sylvatic interface, represented by areas within a zoological park. Sampling was conducted across ten municipalities (Barbosa, Bebedouro, Castilho, Cardoso, Guarulhos, Igaratá, Matão, Pindamonhangaba, São Paulo, and Santa Rita do Passa Quatro) and encompassed two vertical strata, ground level and tree canopy, to capture Culicidae diversity and potential habitat-associated ecological variation (**Figure 1**; **Supplementary Table 1**).

For viral metagenomic analysis, specimens were organized into 45 monospecific pools. Detailed metadata for each pool, including collection site, species, number of individuals, and vertical strata, were systematically recorded to ensure experimental traceability (**Supplementary Tables 1**, **2**).

### 2.2 RNA extraction, cDNA synthesis and sequencing

Total RNA was extracted from the pooled mosquito samples using the Maxwell^®^ 16 Viral Total Nucleic Acid Purification Kit (Promega, Madison, WI, USA) following the manufacturer’s instructions. Genomic libraries were prepared using the Nextera XT DNA Library Preparation Kit (Illumina, San Diego, CA, USA). Sequencing was performed on the Illumina MiSeq platform with paired-end reads (2x 150 bp).

### 2.3 Metagenomic analysis

Raw sequence reads were subjected to quality assessment using FastQC v0.12.1. Adapter sequences and low-quality regions were trimmed using Trimmomatic v0.39 (Bolger et al., 2014). To deplete host-derived reads, trimmed reads were mapped against a Culicidae reference genome dataset retrieved from NCBI Genomes (**Supplementary Table 3**) using Bowtie2 v2.5.1 and removed. Ribosomal RNA was removed using SortMeRNA v.2.1. The filtered reads were subsequently assembled *de novo* using RnaviralSPAdes v3.15.5 (Bushmanova et al., 2019) under default parameters and retaining contig larger than 500 bp.

High-level taxonomic classification of the assembled contigs (Eukarya, Bacteria, Archaea, Viruses, or others) was performed using Kraken 2 against the *standard_prebuilt_core_nt_2024-09–04* database (Wood et al., 2019). Finally, similarity searches were performed using BLASTN, BLASTX, and BLASTP against the NCBI GenBank database to confirm taxonomic assignments. Taxonomic classifications were assigned based on the top-hit from BLASTn and BLASTx searches against the NCBI database (BLAST: basic local alignment search tool). Taxonomic lineages were systematically retrieved using the taxonomizr R package (v0.11.1), which allowed for the conversion of Accession numbers into a standardized hierarchical taxonomy.

In order to discriminate between active, replicating exogenous viral genomes from Endogenous Viral Elements (EVEs) - viral genomic fragments integrated into the host genome, we performed a targeted sequence similarity search. The candidate viral contigs identified in our metatranscriptome were queried against a comprehensive *Culicidae* reference genome dataset retrieved from NCBI Genomes (**Supplementary Table 3**) using BLASTn. Sequences were classified as potential EVEs if they exhibited nucleotide identity > 85% with E-value < 10^^-5^ and query cover > 50%. All flagged sequences were excluded from downstream analyses.

To quantify viral abundance, clean paired-end reads from each library were re-mapped against their respective *de novo* assembled contigs using Bowtie2 (v.2.3.5) (Langmead and Salzberg, 2012) under default parameters. Indexing of contigs was performed with bowtie2-build, and the resulting alignment files (SAM format) were converted to BAM, sorted, and indexed using Samtools (v.1.7). Abundance metrics, including the number of mapped reads, average sequencing depth, and percentage of horizontal coverage, were extracted using samtools idxstats and samtools coverage (Danecek et al., 2021). A custom AWK script was employed to consolidate these metrics into a comprehensive abundance matrix. Only viral contigs with a minimum mean depth of 5x and at least 90% coverage after back-mapping were retained.

### 2.4 Ecological analysis

An ecological sample-by-virus matrix was constructed by aggregating the mapped reads for each viral species across samples. The matrix was structured in a wide format (samples in rows, viral species in columns), with missing values filled as zeros. This matrix served as the baseline for all beta-diversity analyses. To account for variations in sequencing effort, the ecological matrix was rarefied using random subsampling without replacement, with the sampling depth set to the minimum total read count observed among samples. Rarefaction was performed using the rrarefy function from the vegan package to ensure direct comparability of viral composition across samples.

Sample dissimilarity was estimated using the Bray–Curtis index calculated from the rarefied matrix. The structure of the virome composition was visualized through Principal Coordinates Analysis (PCoA), allowing for the graphical inspection of similarity patterns based on ecotope and host taxonomy (tribe, genus and species).

The influence of ecotope, host tribe, genus and species, and spatial and technical covariates on virome composition was assessed using Permutational Multivariate Analysis of Variance (PERMANOVA) via the adonis2 function (vegan package) with 999 permutations. To evaluate the hierarchical influence of host classification without collinearity, separate PERMANOVA models were constructed for host tribe, genus, and species. We tested both sequential and marginal effect models to estimate the independent contribution of each factor to the total explained variance (R^2^). Technical variables (total reads per sample) and spatial variables (collection municipality) were included as covariates to control for their potential impact on the observed patterns.

To determine whether the effects detected by PERMANOVA were attributable to differences in multivariate centroids or to dispersion heterogeneity among groups, we conducted an analysis of multivariate homogeneity of groups dispersions (PERMDISP) using the betadisper function. Statistical significance was evaluated through permutation tests (permutest, 999 permutations).

Host tribe, genus and species were treated as host-related variables. Multivariate results were integrated into composite figures, including: (i) PCoA ordinations color-coded by ecotope and host genus, and (ii) bar charts representing the proportion of variance explained (R^2^) by each factor versus the residual fraction. All statistical analyses and visualizations were performed in R (version 4.0), primarily utilizing the vegan, tidyverse, ggplot2, and patchwork packages.

Bipartite interaction networks between mosquito species and identified viral species were constructed using the igraph and tidygraph R packages. To ensure robust network topology, interactions (edges) were included only for viral contigs exhibiting an average sequencing depth >5x. Nodes were assigned to two functional classes: mosquito hosts (classified by taxonomic tribe) and viral species (annotated by viral family). Network visualization was performed using ggraph with a force-directed layout (nicely), where node sizes were proportional to degree centrality and host nodes were colored by taxonomic tribe.

## 3 Results

### 3.1 Metagenomic overview

NGS sequencing yielded 103,714 assembled contigs. Of these, 82.443 (79.49%) were taxonomically assigned, with the majority attributed to Bacteria (53.24%) and Eukaryota (25.11%). Smaller fractions were identified as Viruses (1.0%) and Archaea (0.09%), while 0.045% were classified as ‘others’ (including non-culturable organisms, expression vectors, and synthetic constructs). Notably, 21,271 contigs (20.51%) remained unidentified, reflecting potentially novel or poorly characterized species. This high proportion of unidentified sequences likely reflect from the absence of reference genomes for many of the mosquito species collected in this study, as well as the divergence of the RNA virosphere.

Although viral contigs represent a small fraction of the total assembly, this finding aligns with published metagenomic surveys highlighting bacterial prevalence and the underrepresentation of viruses in reference databases. This discrepancy underscores the necessity of the in-depth characterization of the mosquito-associated virome performed in this study.

The taxonomic survey identified a diverse array of viral species with varying degrees of host and geographic distribution. A total of 919 viral contigs, after excluding retrotransposons, endogenous virus elements and bacteriophage, belonging to 217 viral species were identified across the 45 metagenomic pools (**Supplementary Table 3**). Among these, 87 viral species (40.1%) were detected in at least two independent pools, providing evidence for a subset of viral agents with the potential for cross-species or cross-regional circulation.

Viruses such as *Mika virus* and *Culex Iflavi-like virus* were among the most prevalent, detected in 20 and 19 independent pools, respectively (**Supplementary Table 4**). Despite being identified primarily within the municipality of São Paulo, these viral agents exhibited a broad host range, circulating across four to five different mosquito species, including *Culex chidesteri* and *Aedes scapularis*. In contrast, other lineages exhibited a more cosmopolitan geographic footprint. For instance, *Aedes aegypti Tovirus 2* and *Hanko totivirus 8* were distributed across multiple distinct municipalities (Barbosa, Cardoso, Guarulhos, and Santa Rita do Passa Quatro for the former; Guarulhos, Matão, and Pindamonhangaba for the latter). These lineages occupied a wide ecological niche, being associated with five and four different host species, respectively.

**Figure 2:**
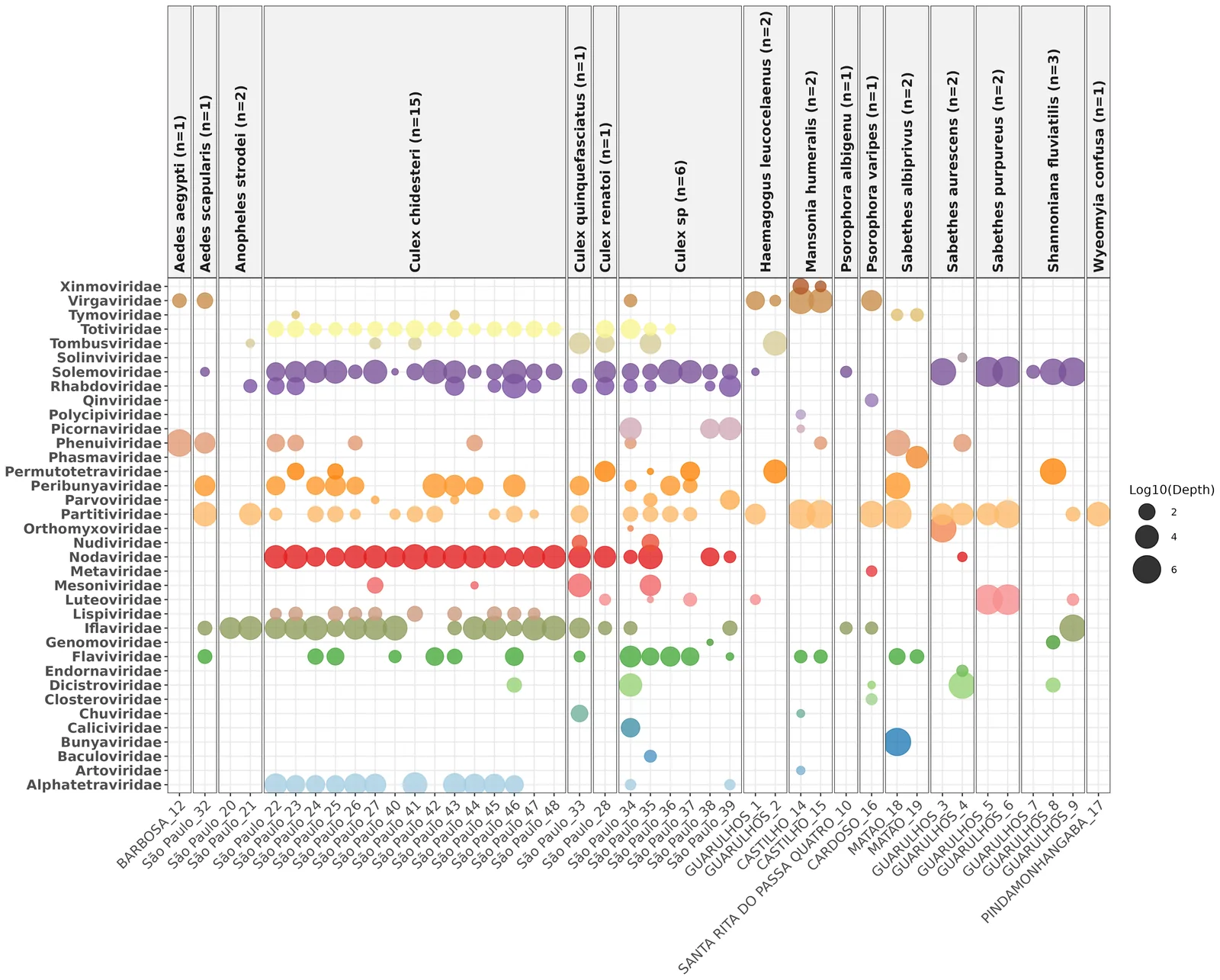
Viral abundance and diversity across mosquito host species. Relative abundance of viral contigs identified in mosquito samples, quantified by the Log10 of sequencing depth. Rows represent viral taxa grouped by family, while columns represent individual mosquito samples.

The taxonomic distribution of the virome across mosquito hosts reveals a highly structured pattern of viral family associations (**Figure 2**). We assigned the viral contigs to at least 37 viral families, most some of which are traditionally associated with arboviruses, such as *Flaviviridae*, *Phenuiviridae*, and *Peribunyaviridae*.Moreover, viruses belonging to the families *Partitiviridae*, *Iflaviridae*, *Solemoviridae*, and *Nodaviridae* were frequently detected, occurring in 12, 12, 9, and 9 mosquito species, respectively. Together these viruses accounted for 47% of the total viral diversity identified in this study (**Figure 2**; **Supplementary Table 4**), suggesting a host-range pattern. In contrast, several viral families exhibited marked hosts specificity, acting as putative specialists restricted to a single mosquito species. For instance, *Closteroviridae* and *Qinviridae* were exclusively identified in *Psorophora varipes*, while *Endornaviridae* and *Solinviviridae* were limited to *Sabethes aurescens*. *Polycipiviridae, Artoviridae*, and *Xinmoviridae* were detected only in *Mansonia humeralis*. Additionally, *Caliciviridae*, *Mesoniviridae*, and *Totiviridae* were found exclusively in *Culex* spp., while *Lispiviridae* was detected solely in *Culex chidesteri*.

### 3.2 Ecology determinant of virome architecture

**Figure 3:**
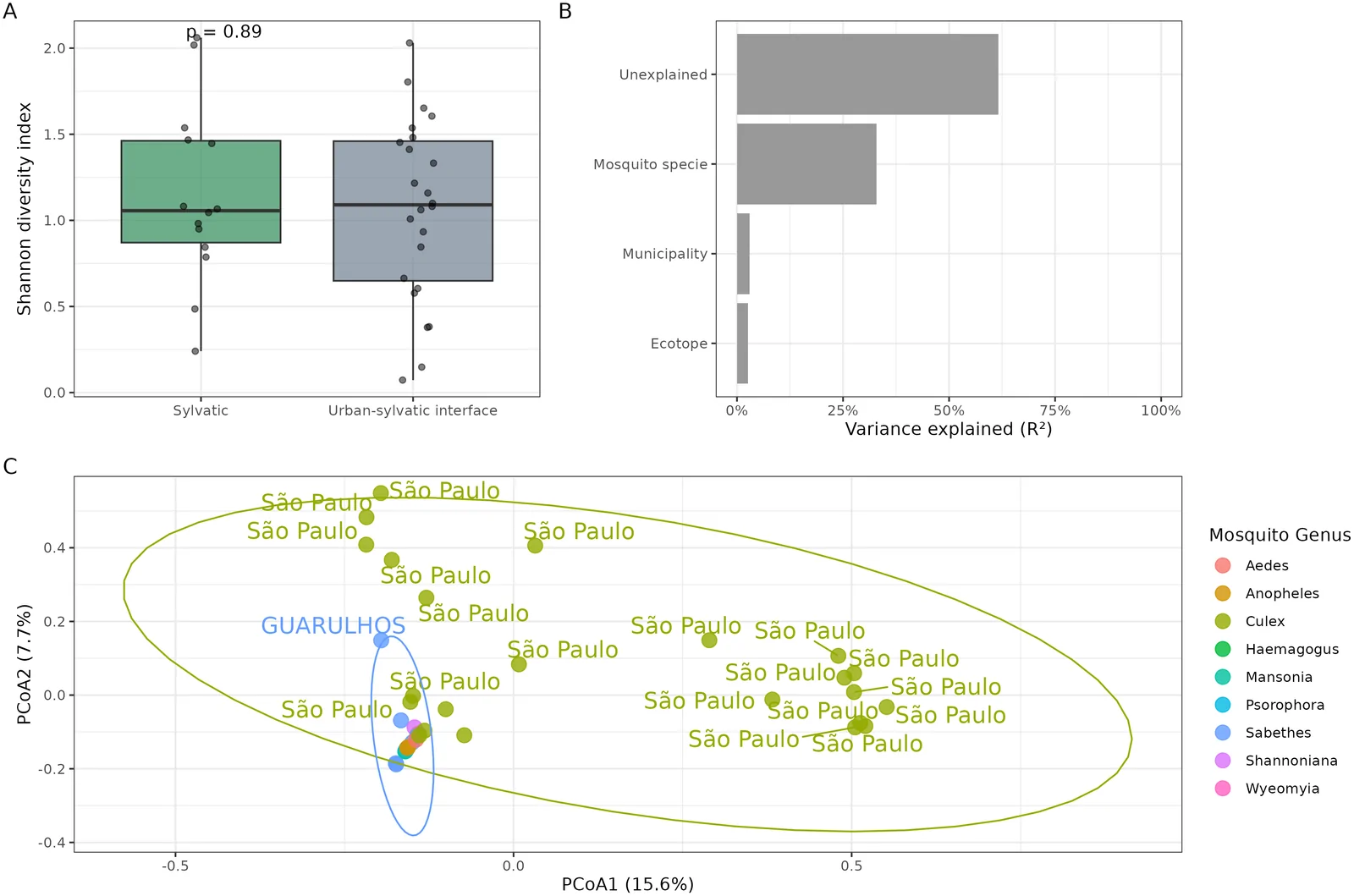
Environmental and host-associated structuring of mosquito viromes. **(A)** Alpha diversity of mosquito-associated viral communities estimated using the Shannon diversity index. Each point represents an individual mosquito pool, and boxplots summarize the median and inter-quartile range. **(B)** Multivariate dispersion of viral communities by mosquito host genus based on Bray–Curtis dissimilarities. Distances of samples to their respective group centroids reveal significant heterogeneity among host species (*betadisper*; permutation test with 999 permutations, *p* = 0.001). **(C)** Principal Coordinates Analysis (PCoA) of mosquito viromes based on Bray–Curtis dissimilarity calculated from rarefied viral abundance matrices.

To assess viral community turnover across mosquito hosts and sampling locations, we performed a Principal Coordinates Analysis (PCoA) based on Bray–Curtis dissimilarity (**Figure 3C**). The first two axes explained 23.3% of the total variance (PCoA1 = 15.6%, PCoA2 = 7.7%) and revealed a predominantly host-driven structure of the mosquito virome. Our results indicate that viral alpha diversity, as measured by the Shannon Index, does not significantly differ at the broad landscape level between sylvatic and urban-sylvatic interfaces (*p* = 0.89; **Figure 3A**). This suggests that anthropization does not lead to a generalized loss of viral complexity. However, this stability at the ecotope level masks significant host-specific patterns; while the environment as a whole maintains similar diversity levels, specific sylvatic specialists (e.g., *Sabethes* spp. and *Shannoniana fluviatilis*) harbor a significantly higher richness of viral species compared to urban-adapted vectors.

Thus, high viral diversity is a trait preserved by specific forest-dependent host species rather than a uniform characteristic of the forested habitat. In contrast, samples associated with *Aedes*, *Haemagogus*, *Psorophora*, and *Sabethes* were positioned on the positive side of PCoA1 and displayed greater dispersion along PCoA2, reflecting increased viral heterogeneity and higher multivariate dispersion (**Figure 3C**). Samples from Guarulhos occupied an intermediate position in the ordination space, suggesting transitional viral assemblages influenced by both urban-associated *Culex* populations and more diverse sylvatic or peri-urban vectors.

The ordination patterns were statistically supported by PERMANOVA analyses using Bray–Curtis dissimilarities. In a multifactorial model including host taxonomy, ecotope, municipality, and sequencing depth, the overall model explained a significant proportion of variance (R^2^ = 0.570, F = 1.985, *p* = 0.001), with host taxonomy and municipality emerged as the primary significant predictor of viral community composition, while sequencing depth showed a marginal effect (R^2^ = 0.030, *p* = 0.057).

Host taxonomy proved to be the major determinant of viral community structure, displaying a distinct taxonomic resolution gradient. When evaluated at the finest level, host species accounted for the highest proportion of unique variance (PERMANOVA, R^2^ = 0.329, F = 2.38, *p* = 0.001). Broadening the resolution to host genus and tribe maintained high statistical significance, though with progressively lower explanatory power (R^2^ = 0.171, F = 1.81, *p* = 0.001 for genus; R^2^ = 0.134, F = 2.13, *p* = 0.001 for tribe). Conversely, spatial structuring (municipality) showed the opposite trend, explaining a minimal fraction at the species level (R^2^ = 0.029, *p* = 0.001) but surpassing host contribution at the tribe level (R^2^ = 0.147, *p* = 0.001) (**Supplementary Table 5**).

Municipality and ecotope did not significantly explain virome variation under this full model. Given the primary influence of host-related factors, a reduced model incorporating host species and ecotope was further evaluated. In this simplified framework, host species accounted for a substantially larger proportion of the variance (R^2^ = 0.329, F = 2.03, *p* = 0.001), whereas ecotope and municipality contributed a smaller but statistically significant effect (**Supplementary Table 6**). Notably, the majority of the variance remained unexplained (68.5%), pointing to additional unmeasured microenvironmental or stochastic drivers (**Figure 3B**).

The influence of environmental stratification on virome composition thus appears to be model-dependent; while ecotope was a significant predictor in the reduced model, its effect was largely masked when geographic location (Municipality) was included. This suggests that while the ecotope exerts a subtle modulation on viral diversity, its signature is secondary to the stronger geographic and host-specific drivers that dominate the virome architecture. Collectively, these findings demonstrate that mosquito host species are the primary determinant of virome composition, while ecological and spatial factors act as secondary modulators of viral community structure.

### 3.3 Network hubs in viral sharing

**Figure 4:**
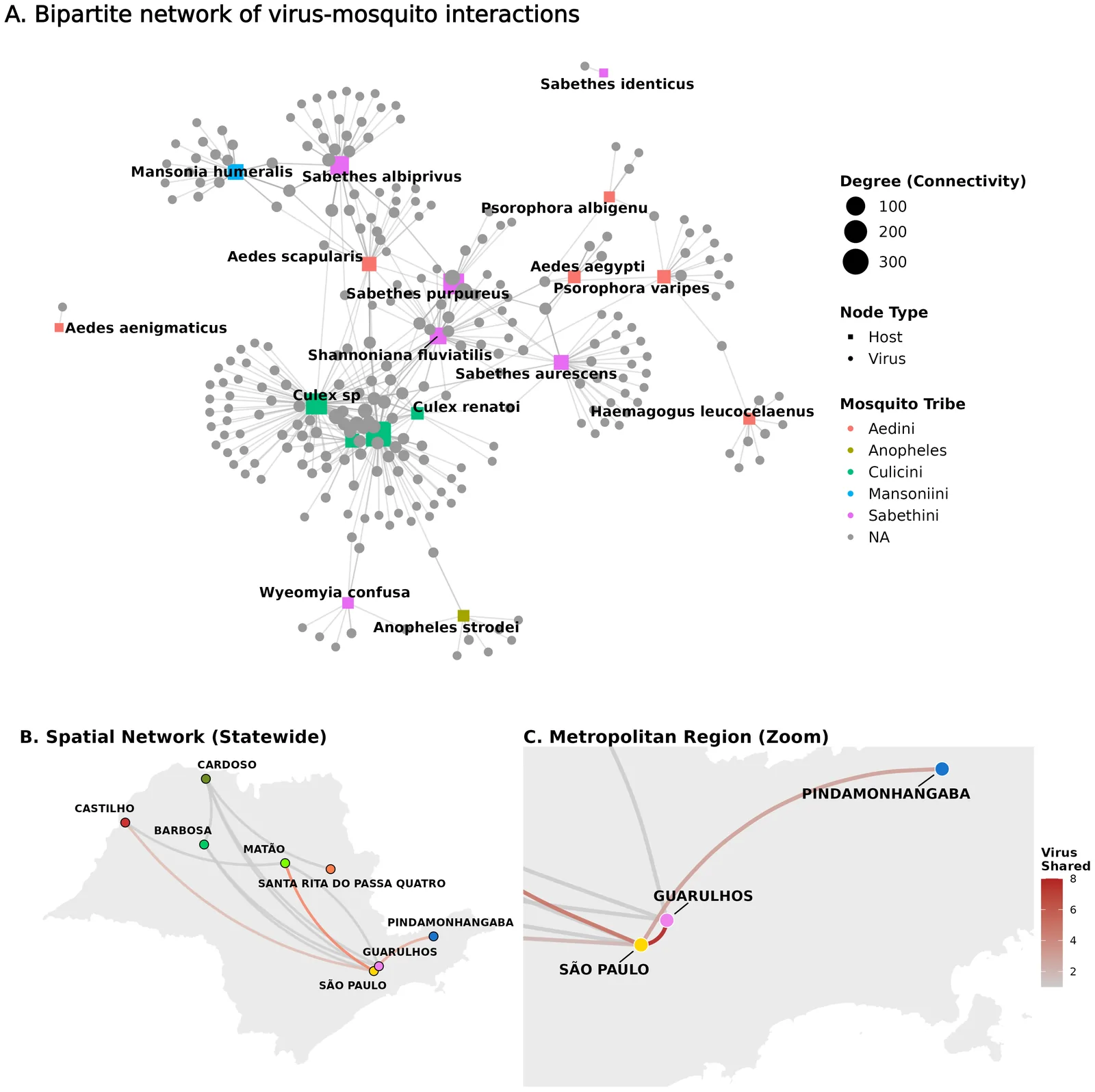
Viral sharing networks across mosquito host species and geographical locations. **(A)** Bipartite network of virus-mosquito interactions. The graph visualizes the connectivity between mosquito host species (squares, colored by mosquito tribe) and identified viral species (gray circles). Edges represent a positive viral detection with a sequencing depth >5x. The size of the viral nodes is proportional to their degree (number of host connections); larger nodes indicate generalist viruses shared across multiple host species, while smaller nodes represent specialist viruses restricted to a single host. **(B)** Statewide spatial network of shared viruses across sampled municipalities. Nodes represent sampling sites, and connecting edges indicate the presence of at least one shared viral species between two locations. Edge color gradients (from gray to red) indicate the absolute number of shared viral species, highlighting geographical hubs of viral circulation. **(C)** Zoomed-in view of the Metropolitan and Paraíba Valley regions, emphasizing the high viral connectivity among São Paulo, Guarulhos, and Pindamonhangaba.

To further explore the ecological structure of the assembled virome, we constructed a bipartite interaction network. This topological analysis allows for the visualization of viral sharing patterns (connectivity) versus host specificity (**Figure 4A**). The network has a modular, star-like structure in which most viruses connect to just one host (degree = 1). This indicates that host-specific viruses, rather than a shared environmental viral pool, drive the distinct virome profiles observed. The genera *Culex* (notably *Cx. chidesteri*, *Cx. quinquefasciatus*, and *Culex* sp.) emerges as the most prominent network hub, exhibiting the highest node degree and capturing a vast repertoire of exclusive viral species. While these species dominate the network’s complexity, a more interconnected sub-component is observed among *Aedes aegypti*, *Psorophora* spp., and *Sabethes* spp. Crucially, species such as *Sabethes albiprivus* and *Aedes scapularis* act as central connectors, sharing viral nodes with multiple taxa. This topological centrality suggests these species may function as bridge vectors, facilitating viral maintenance across different ecological niches. Conversely, *Aedes aenigmaticus* and *Sabethes identicus* form disconnected or satellite modules. Their viral partners do not share edges with the main network component, reinforcing the hypothesis of highly distinct, insulated viromes for these populations. Multi-host viral nodes are sparse and primarily link phylogenetically or ecologically related hosts, suggesting that cross-species viral transmission in this ecosystem is constrained by host phylogenetic distance and ecological barriers.

To evaluate whether geographical proximity connectivity influences viral distribution, we constructed a spatial sharing network across the nine sampled municipalities in São Paulo State (**Figures 4B, C**). Viral sharing across distant inland municipalities (e.g., Castilho, Cardoso, Matão, and Santa Rita do Passa Quatro) remained relatively low to moderate, with most long-distance pairwise site connections mediated by a limited subset of broadly distributed viral species (**Figure 4B**). In contrast, spatial connectivity was markedly pronounced within highly urbanization-impacted regions (**Figure 4C**). A prominent sharing hub was identified between the adjacent municipalities of São Paulo and Guarulhos, which exhibited the highest degree of viral overlap (up to 8 shared viral species, depicted by thick, dark-red edges). Notably, spatial isolation alone does not explain these patterns, statistical testing revealed no significant association between geographic distance and viral sharing or community dissimilarity (Mantel test: r = -0.02, *p* = 0.535 for shared viruses; r = 0.017, p = 0.461 for Jaccard dissimilarity; **Supplementary Figures 3A, B**). This lack of distance decay suggests that long-range viral dispersal is not driven by continuous spatial diffusion, but rather facilitated by anthropogenic factors, such as major transportation corridors and high human mobility centered around the São Paulo–Guarulhos hub, which promote the passive transport of vector mosquitoes or host movement across the state.

## 4 Discussion

Characterization of mosquito viromes across the sylvatic–urban gradient facilitates both the discovery of a diverse reservoir of previously undescribed viruses maintained in natural ecosystems and the identification of emerging pathogenic threats to human populations. In addition to the surveillance of high-consequence agents, the detection of insect-specific viruses (ISVs) is critically important, as these viruses may substantially influence the replication dynamics of co-infecting arboviruses through mechanisms such as superinfection exclusion and immune priming. Elucidating the spatial distribution and host specificity of viral communities in field-collected mosquitoes therefore yields fundamental insights into the transmission dynamics of viruses of public health significance. To date, few studies have characterized the mosquito virome in Brazil, with existing research primarily focused on a restricted range of species across diverse biomes, including the Cerrado, Caatinga, Pantanal, Amazon, and the Atlantic Forest of the Northeast and Southeast regions (Lara Pinto et al., 2017; Maia et al., 2019; Scarpassa et al., 2019; Da Silva Ferreira et al., 2020; Ribeiro et al., 2020; Da Silva et al., 2021; Da Silva Neves et al., 2021; Aragão et al., 2023; Da Silva et al., 2024).

Our investigation identified 919 viral sequences classified into 37 distinct families from sylvatic mosquito populations within the São Paulo municipalities. We identified a high diversity of viruses belonging to families traditionally associated with pathogenic arboviruses, including *Phenuiviridae*, *Flaviviridae*, *Rhabdoviridae*, and *Peribunyaviridae*. The widespread detection of these viral families across various mosquito species and vertebrate hosts highlights a broad host permissiveness, which likely facilitates their capacity for interspecies transmission and environmental persistence. Notably, and consistent with recent large-scale metagenomic surveys of field-caught mosquitoes, no known high-consequence pathogenic arboviruses were detected in our dataset within the sensibility of our sampling and sequencing depth (Mirza et al., 2024; Guimarães et al., 2025).

The absence of detectable pathogens may result from a dominant native virome in mosquito populations. Mosquitoes with an established, non-pathogenic viral community often display reduced vector competence due to viral interference. Resident viruses can inhibit the acquisition, replication, and transmission of exogenous arboviruses (Öhlund et al., 2019). This interference typically occurs through superinfection exclusion or constitutive activation of the mosquito’s innate antiviral defenses (Laureti et al., 2020). Thus, the resident virome may function as a biological barrier that limits the ability of mosquito populations to act as long-term reservoirs or competent vectors for emerging viruses. On the other hand, detection sensitivity is intrinsically constrained by sequencing depth and viral load. Across all sequencing libraries, a mean of 595,754 reads were generated per sample (range: 181,325 - 1,087,562) (**Supplementary Table 2**). Although this sequencing depth was sufficient to characterize the dominant viral community and identify low-abundance ISVs, we acknowledge that low-prevalence or low-titer human pathogens (e.g., arboviruses circulating at sub-threshold levels in vector pools) may remain below the limit of detection of unbiased metagenomic workflows.

Our findings reveal an association of virome composition by mosquito species, with geography (defined as the municipality of collection and ecotype) exerting a significant, albeit secondary, influence. These results underscore that mosquito species act as the primary biological determinant in shaping viral communities across different regions of São Paulo state, aligning with previous studies that emphasize host-specific viral signatures (Shi et al., 2019; Batson et al., 2021). Notably, this host-driven structuring was evident even at the intra-generic level, where distinct viral communities were maintained between different species within the same genus.

The majority of identified viruses exhibited either strict host specificity or a marked host preference, characterized by significantly higher abundance in one particular host. Our PERMANOVA analysis across host taxonomic ranks demonstrates an escalation in host-driven virome structuring. While higher taxonomic levels such as tribe and genus significantly influenced viral composition, they accounted for only 13.3% and 17.1% of the total variance, respectively (**Supplementary Table 5**). In contrast, host species identity emerged as the definitive determinant, explaining 32.9% of the virome variation (R^2^ = 0.329, *p* = 0.001, **Supplementary Table 6**). This marked increase in explanatory power from genus to species levels highlights a potent species-specific barrier. It implies that the mosquito virome is not merely a reflection of broad phylogenetic lineages but is finely tuned by the unique biological or ecological traits of each species (Feng et al., 2022).

The lack of a continuous distance-decay pattern across São Paulo State indicates that spatial distance plays a secondary role in shaping mosquito RNA viral communities. Traditional spatial ecology models assume that biological communities diverge with physical distance due to natural dispersal limitations. However, our Mantel analysis revealed no significant correlation between spatial distance and either viral species sharing (r = -0.02, p = 0.535) or compositional dissimilarity (r = 0.017, p = 0.461; **Supplementary Figure 3**). These findings highlight that long-range viral dispersal across the state is not constrained by physical distance or geographic barriers, but is predominantly dictated by host species specificity coupled with human-mediated vector movement. Passive transport of mosquitoes via vehicles and cargo along major transport corridors likely drives long-distance jump-dispersal events, connecting inland municipalities to highly connected metropolitan hubs like São Paulo and Guarulhos (up to 8 shared viral species). Ultimately, this demonstrates that characterizing the mosquito virome serves as a powerful proxy to uncover vector movement and spatial ecology, showing how human infrastructure and host evolutionary identity jointly structure viral landscape dynamics across broad geographic scales.

While most ISVs and persistent viruses are thought to constitute a stable ‘core virome’ within a species (Konstantinidis et al., 2022a), our data suggest that this stability is modulated by a complex interplay of factors and strongly shaped by host-species. As proposed by De Coninck and Matthijnssens (2024), the mosquito virome concept must be approached with nuance, as it is influenced by host biology, geographic region, dietary sources, microbiota, and environmental fluctuations (De Coninck and Matthijnssens, 2024; Koh and Saleh, 2024). Consequently, while the host species is the dominant driver, the observed geographic variations indicate that viral diversity is also shaped by local ecological contexts, suggesting that findings from specific populations should be extrapolated to global scales with caution (Li et al., 2015; Shi et al., 2016). Interestingly, the lack of significant variation between canopy and ground strata further reinforces the hypothesis that intrinsic host biology, rather than vertical niche partitioning, is the overriding factor in defining the mosquito virome architecture in these forested areas (Guimarães et al., 2025).

The transition of viral agents from sylvatic reservoirs to urban cycles is driven by the ecological interface between distinct host populations, facilitated by the fragmented landscapes of São Paulo. Our bipartite network identifies bridge species, such as *Aedes scapularis* and *Sabethes albiprivus*, as critical connectors that bridge the gap between forest and peri-urban vectors like *Aedes aegypti*. This breakdown of ecological insulation is reflected in the high amino acid identity (AAI >90%) of families such as *Phenuiviridae* and *Peribunyaviridae*, which circulate across species boundaries with minimal adaptive barriers. In contrast, the high novelty (AAI <40%) and strict modularity of the *Culex*-associated virome suggest a sequestered reservoir of ISVs, identifying specific taxa as priority nodes for genomic surveillance in the forest-urban interface.

The disparate distribution of viral families across the mosquito community reveals a complex interplay between viral generalists and host-restricted specialists, providing empirical evidence for the theory of RNA virus evolution. The high frequency of families such as *Partitiviridae, Iflaviridae, Solemoviridae*, and *Phenuiviridae*, which together constitute 47.0% of the total viral diversity and span up to 12 different host species suggests a remarkably broad host-range pattern. From an ecological perspective, these viruses act as communal vOTUs that permeate the taxonomic boundaries of the mosquito community. This ecological breadth is further supported by a positive association between host range and sequence conservation where families with the widest distribution also exhibit high amino acid identity (AAI >90%). Their prevalence across phylogenetically distant genera, such as *Aedes*, *Culex*, and *Sabethes*, implies high adaptive plasticity or the utilization of conserved cellular receptors.

This widespread occurrence likely reflects frequent horizontal transfer events facilitated by sympatry, where shared nectar-feeding sites or communal larval habitats serve as environmental hubs for viral exchange. Beyond simple contact, the broad host-range potential observed in these communities is sustained by multifaceted maintenance cycles where cross-species transmission appears to occur more frequently than long-term virus-host co-divergence. In nature, viruses are horizontally transmitted among mosquito populations sharing the same ecological niche, even between distantly related taxa, through mechanisms such as shared larval habitats or communal feeding sites (Ortiz-Baez et al., 2022; Li et al., 2023).

The low genetic divergence observed in these multi-host families indicates that these generalists circulate with minimal adaptive barriers. Such frequent horizontal transfer events across aquatic and terrestrial ecosystems position arthropods as the engine room of RNA virus evolution, playing a crucial role in the emergence of novel viruses in closely associated organisms, including vertebrates (Dudas and Obbard, 2015; Dolja and Koonin, 2018). This evolutionary engine is fueled by the constant exposure of mosquitoes to diverse viral pools in sympatric settings, providing the raw material for rapid diversification. Consequently, the conserved viral lineages shared between *Aedes* and *Sabethes* in our study provide empirical evidence of high host-switching potential, where low genetic divergence serves as a precursor for the emergence of pathogens into the urban environment.

Our study reveals that the architecture of the mosquito virome in São Paulo State is predominantly shaped by host identity at species level, indicating strong evolutionary specialization and stable ecological associations, though this interpretation is tempered by key limitations: the opportunistic sampling strategy and significant temporal asynchrony between interior collections (October 2019–March 2020) and capital collections (May–December 2020) introduce potential confounding from seasonal and epidemiological variation that future standardized longitudinal studies must resolve.

Despite these caveats, host factors consistently explained the largest proportion of viral community variance, with environmental stratification exerting only a secondary, often-masked influence, suggesting the virome is remarkably resilient to local ecological shifts. The detection of a vast array of viral lineages from conserved pathogens to divergent novel viruses underscores the Neotropical sylvatic environment as a major reservoir of viral dark matter, while network analysis revealed both generalist viruses with high connectivity across mosquito species (suggesting horizontal transmission or host-switching) and specialist lineages with restricted distributions (implying vertical transmission and co-speciation). Collectively, these findings emphasize that species-level host-pathogen resolution is critical for predicting viral ecology and spillover potential in rapidly changing landscapes.

## 5 Conclusion

This study substantially expands the current understanding of viral diversity in Neotropical mosquitoes, revealing a large reservoir of poorly characterized viruses, particularly within predominantly sylvatic genera. The strong host-driven structuring of the virome demonstrates that intrinsic mosquito biology is the primary determinant of viral community composition, whereas environmental and spatial factors play secondary roles. The highly modular virus–host network, dominated by host-restricted specialists, highlights ecological and phylogenetic barriers that constrain viral sharing across species.

The absence of high-consequence arboviruses, despite the detection of several medically relevant viral families, is consistent with the hypothesis that the native virome may contribute to a biological barrier against exogenous infections. Although these findings should be interpreted in light of temporal sampling and sequencing depth limitations, they reinforce the importance of host identity in shaping viral ecology. Together, these results highlight the importance of integrative metagenomic and ecological frameworks for understanding viral dynamics and predicting arbovirus emergence in rapidly changing environments.

## Data Availability

The datasets presented in this study can be found in online repositories. The names of the repository/repositories and accession number(s) can be found in the article/**Supplementary Material**.
