# Supplementary material for: RNA virome comparison between sylvatic and urban-interface mosquitoes from Southeastern Brazil
Source: Front Cell Infect Microbiol. 2026 Sep 4;16:1894867. doi: 10.3389/fcimb.2026.1894867 (PMC13585534; doi:10.3389/fcimb.2026.1894867)
Supplement: Supplementary file 1 [file Table2.docx]

**Supplementary Figure 1: Distribution of amino acid identity of viral contigs by family and mosquito host.** Boxplots show the percentage of amino acid identity (BLASTx against NCBI NR database) for viral contigs mapped to specific families. Each point represents a distinct viral contig, color-coded by the mosquito species from which it was assembled. The vertical dotted lines at 40% and 70% identity serve as arbitrary reference thresholds for potential novel taxonomic ranks (e.g., genus or species level divergence). Families are ordered by their median identity values. The plot reveals a wide range of conservation, from highly conserved viruses in Orthomyxoviridae and Mesoniviridae (>90% identity) to deeply divergent, putative novel viruses in families such as Alphatetraviridae and Artivuridae (<40% identity).

**Supplementary Figure 2:** Bipartite network of virus-mosquito interactions. The graph visualizes the connectivity between mosquito host species (squares, colored by mosquito tribe) and identified viral species (gray circles). Edges represent a positive viral detection with a sequencing depth >10x. The size of the viral nodes is proportional to their degree (number of host connections); larger nodes indicate generalist viruses shared across multiple host species, while smaller nodes represent specialist viruses restricted to a single host.

**Supplementary Figure 3:** Relationship between geographic distance (km) and viral community composition across pairwise sampling locations in São Paulo State. (A) Pairwise number of shared viral species. (B) Pairwise Jaccard dissimilarity index of viral composition. Solid trend lines represent fitted regression models, with shaded areas depicting 95% confidence intervals. Mantel test statistics (r and p-values) indicate no statistically significant correlation between spatial distance and viral sharing or community dissimilarity.
